# Influence of Fused Filament Fabrication Strategy on Polyamide Properties

**DOI:** 10.3390/ma18225241

**Published:** 2025-11-19

**Authors:** Marta Beata Krawczyk, Marcin Andrzej Królikowski, Kamil Urbanowicz

**Affiliations:** Faculty of Mechanical Engineering and Mechatronics, West Pomeranian University of Technology in Szczecin, 70-310 Szczecin, Poland; marta.krawczyk@zut.edu.pl (M.B.K.); marcin.krolikowski@zut.edu.pl (M.A.K.)

**Keywords:** additive manufacturing, fused filament fabrication, nylon, polymer testing, tensile test of thermoplastics

## Abstract

This study investigates the influence of Fused Filament Fabrication (FFF) parameters on the properties of polyamide (PA, Nylon™) parts, which are valued for their excellent mechanical properties in additive manufacturing. The parameters examined include infill structure (diagonal and honeycomb), infill density (60%, 80%, and 100%), and sample orientation (0°, 45°, and 90°) relative to the build plate. Filaments from five manufacturers were tested, with injection-molded samples serving as references. Standard tensile strength tests were performed. The results indicate that the 0° orientation yielded the highest tensile strength, while the 45° and 90° orientations exhibited distinct behaviors associated with the geometry of additive manufacturing. The highest Young’s modulus was obtained for solid infill at 0° orientation. Although infill structure had a smaller effect, the honeycomb pattern provided more stable and superior mechanical properties at higher infill densities. The study compared filaments from different manufacturers, identifying two that met the tensile strength requirements for telerehabilitation device case prototypes.

## 1. Introduction

Additive manufacturing (AM) is a key technology highlighted by the 4th Industrial Revolution, driven by the paradigm of mass, personalized production. This technology encompasses a broad range of materials, from liquid crystal polymer resins and thermoplastics to metals [1]. Among the various AM techniques, Fused Deposition Modeling (FDM) also known as Fused Filament Fabrication (FFF), Modeling Extrusion (MEX), Fused Layer Manufacturing (FLM), or 3D printing has become one of the most widely used methods [2,3,4]. The advantages of FFF include the ability to produce complex shapes efficiently, reducing both time and cost in prototype and small series production [3].

Common thermoplastic materials used in the FFF process include ABS (acrylonitrile butadiene styrene), PMMA (poly (methyl methacrylate)), PCL (poly (ε-caprolactone)), PLA (polylactic acid), and PC (polycarbonate) [3,5,6]. Polyamide (PA), commonly known as Nylon™, is a biocompatible polymer with excellent printability and processability in FFF. It offers outstanding mechanical properties such as fatigue endurance, impact resistance, vibration absorption, a low friction coefficient, and flexibility at low temperatures, in addition to excellent chemical resistance. These properties make Nylon™ suitable for a wide range of applications across industries such as automation, aviation, automotive, and even medical implants [3,7,8,9,10]. However, one of the main drawbacks of PA is its tendency to absorb moisture, which can significantly reduce its strength and stiffness. Jia, N. et al. [11] studied the effects of conditioning methods on the mechanical properties of PA6 as a function of temperature and relative humidity. They showed that appropriate sample conditioning offered the possibility of optimizing its mechanical properties. Surface modification to reduce water adsorption was investigated by Miguel, M. et al. [3]. In their work, they noted that coating polyamide components fabricated by FDM with polyurethanes had a positive effect on reducing water adsorption and external porosity with a minor reduction in strength and stiffness. Modification of polymeric materials using metallic coatings was explored by Verdi, D. et al. [9]. Their work aimed to expand the potential applications of polyamide 12 (PA12) as a result of improved electrical properties and wear resistance. They showed, among other things, that the LPCS process parameters play a key role in determining the thickness, adhesion and homogeneity of the metallic layer and that the metallization process affects the thermal and physical properties of the PA12 substrate, causing local increases in crystallinity and plastic deformation resulting from the heat and kinetic energy of the metallic particles. Elements produced using the FFF technique are primarily used for visualization, presentation or educational models, and for assembly or replacement parts. However, functional components made with FFF are increasingly being applied in more demanding fields, such as the production of turbine blades, rapid tooling, and rapid casting of aluminum parts, where FFF-produced components serve as patterns or molds. Additionally, FFF techniques hold great promise for manufacturing biomedical devices, including implants, prostheses, and tissue engineering scaffolds [2,3,4,12]. For example, Rahim, T.N.A.T. et al. produced a PA12 filament with a bioceramic filler for use as a biomedical material. They showed that the addition of zirconium oxide (ZrO_2_) and hydroxyapatite (HA) at 10–30 wt.% could improve the stiffness and strength of the polyamide, while reducing its flexibility and impact resistance. These results show that the developed composite may find applications in biomedicine, especially in implants subjected to low mechanical loads.

Discussed applications impose specific requirements, making it crucial to optimize the FFF process to maximize its advantages and mitigate limitations. Understanding how parts behave under mechanical loading is essential for assessing their suitability for specific applications [2,7].

A significant amount of research has been dedicated to investigating the impact of FFF process parameters on the properties of the final product. It is important to note that these parameters can be categorized into three main groups: (a) slicing parameters—such as layer thickness, nozzle diameter, flow rate, deposition speed, infill density, and infill pattern [6,7,12,13]; (b) build orientation—horizontal, vertical, lateral, or angled [4,6,12,13]; and (c) temperature conditions—environmental temperature, extrusion temperature, and platform temperature [2,10,12]. The works [5,14,15,16] summarize the most important conclusions drawn from the above groups of process parameters, or their compilation. However, most studies have focused on commonly used materials like ABS and PLA, while the literature on the influence of FFF parameters on the mechanical properties of Nylon™ (PA) remains limited.

The main objective of this research was to compare the properties of PA supplied by different manufacturers and select the best one for use in the production of prototypes for telerehabilitation device enclosures. Since these new devices were at the prototype stage, it was assumed that the most effective solution would be additive manufacturing using the FFF method for small-batch production of PA enclosures. Given the wide range of filament manufacturers, there may be significant differences in material properties. In addition, the FFF process can cause geometric and structural inconsistencies in the manufactured parts [17,18,19]. Another important aim of this study was to investigate how both material properties and FFF process parameters such as build orientation, infill density, and infill pattern, affect the tensile strength of polyamide polymer supplied by five different manufacturers.

## 2. Materials and Methods

**Figure 1:**
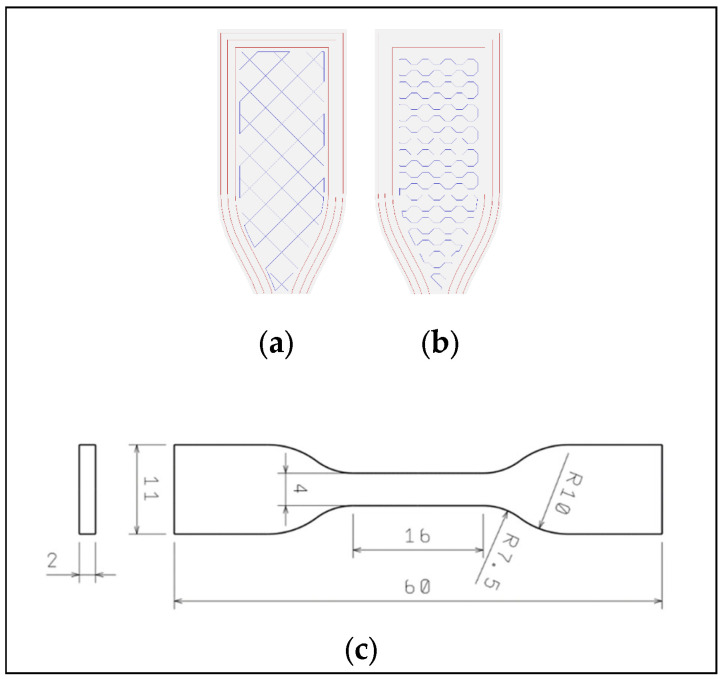
Types of infill generated (middle layer layout): (**a**)–diagonal, (**b**)–honeycomb, (**c**) specimen dimensions.

The commercial polyamide materials in the form of 1.75 mm diameter filaments, sold under the trade name Nylon, were used in this study. These materials originated from five different manufacturers and were purchased in their natural, undyed form. To verify the manufacturers’ claims regarding the composition of PA12 filaments, ATR-FTIR spectroscopy was conducted at a later stage of the research. Specimen models for tensile testing were designed using Solidworks 2016 software (Dassault Systèmes, Paris, France) in accordance with EN ISO 527-2:2012 [20]. For comparison, additional samples were produced using the injection molding method. The samples were manufactured by additive manufacturing (AM) using the material extrusion (MEX) process, specifically layer plastic deposition (LPD) technology. A Zortrax M200 printer (Zortrax, Olsztyn, Poland) equipped with an optional closed chamber and direct extruder was used. Prior to printing, the filaments were dried at 80 °C for 42 h to eliminate moisture. The device was thermally stabilized and passively heated by the printing bed, for one hour before initiating the process. The specimen models were imported into Zortrax Z-Suite slicing software (Zortrax, Olsztyn, Poland). The selected specimens for tensile testing had dimensions 60 mm × 11 mm × 2 mm with a tested area of 16 mm × 4 mm. Raft adhesion mode was applied to ensure proper attachment to the build plate. The printing speed was set to 30 mm/s, and a layer thickness of 0.1 mm was applied to all samples. Retraction distance, retraction speed, wall thickness, bottom and top layers, and infill speed were set according to the default settings recommended by the slicer for PA12 non-Zortrax filaments. Nozzle and bed temperatures were adjusted according to the mean temperature recommended by the filament manufacturers. Nozzle temperature was set to 260 °C and bed temperature to 100 °C. Different deposition strategies were applied. Each batch of samples was printed in three orientations relative to the tensile axis and machine bed: 0°, 45°, and 90°. The infill densities used were 60%, 80%, and 100%, with diagonal and honeycomb infill structures (Figure 1).

**Figure 2:**
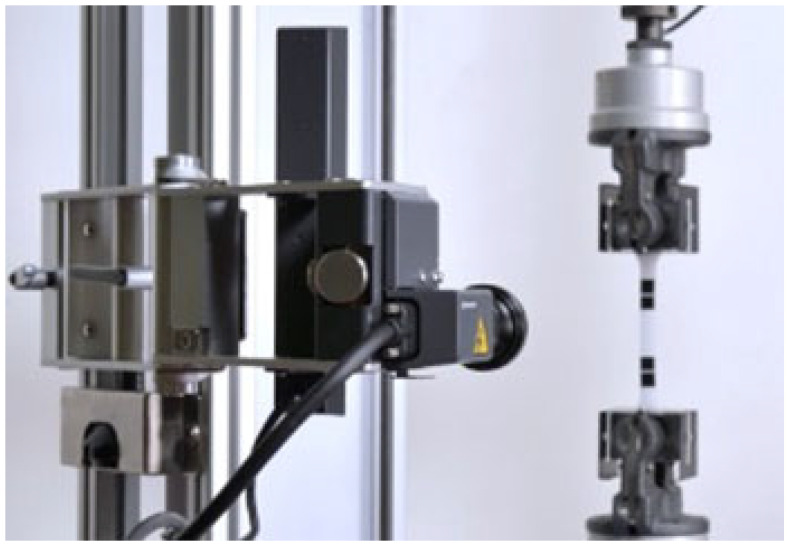
Shimadzu AG-Plus 10kNX testing machine with a non-contact extensometer.

For each combination of parameters, 10 specimens were produced. Tensile tests were performed according to the ISO 527-1/2:2025 standard [20,21] using a Shimadzu Autograph AG-X Plus universal testing machine (Shimadzu, Tokyo, Japan) (Figure 2). Type 5A test specimens were used in this study.

The machine was equipped with a 1 kN load cell, a TRViewX non-contact video extensometer, and pneumatic grips with a rubber coating. TrapeziumX software (Shimadzu, Tokyo, Japan) was used for data acquisition and processing. The tests were carried out at room temperature. A crosshead speed of 1 mm/min was applied until an elongation of 1% was reached, after which the speed was increased to 5 mm/min until fracture. Tensile stress and elongation at break were determined from stress–strain curves, while Young’s modulus was calculated from the slope of the stress–strain curve in the strain range of 0.05–0.25%. The chemical composition was analyzed using a Tensor 27 FTIR spectrometer (Bruker, Ettlingen, Germany) equipped with an ATR unit. Measurements were performed in a single reflection setup with a monocrystalline diamond crystal, in the range of 400–4000 cm^−1^. Thermogravimetric measurements were carried out on a TG-DSC Q600 device (TA Instruments, New Castle, DE, USA). The samples were heated to 700 °C at a heating rate of 10 K/min in an atmosphere of synthetic air. Microscopic examination of the materials fractures after the tensile test was conducted using a 4K Keyence VHX-7000 microscope (Keyence, Osaka, Japan). A two-way analysis of variance (ANOVA) without replication was conducted to determine the significance of process variables on Young’s modulus.

## 3. Results

**Figure 3:**
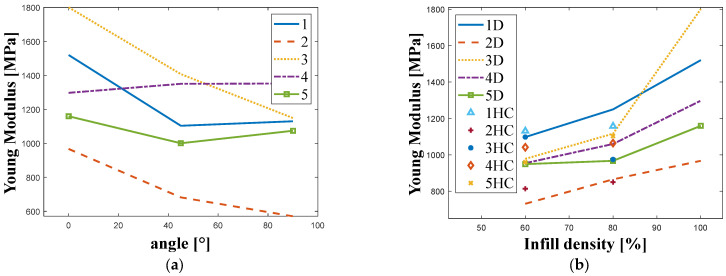
Plots of Young’s modulus obtained for the tested manufacturers for: (**a**) Different manufacturing angle. (**b**) Different infill density and infill structure. ((1–5)—manufacturer; HC—honeycomb; D—diagonal).

The results of the tensile tests for each combination of material, infill density, and orientation angle are summarized in Table A1 (found in the Appendix A). The average values of Young’s modulus, tensile strength, and elongation at break are presented, along with the standard deviation calculated from the samples within each batch to ensure reliable results. The tensile test results are also presented in the form of graphs. Figure 3 shows the relationship between Young’s modulus and the manufacturing angle (Figure 3a) and the infill density for different PA12 materials (obtained from different manufacturers) and infill patterns (Figure 3b).

Based on the chart presented in Figure 3, it can be concluded that the results for samples made from the material supplied by Company 2 were consistently the lowest, regardless of sample orientation. In the other cases, the highest Young’s modulus values were obtained for the 0° orientation, except for the material supplied by Company 4. For the 45° and 90° orientations, the calculated Young’s modulus values were lower than those for the 0° orientation. A similar pattern was observed for samples made from materials from Companies 1 and 5. These results are consistent with those reported by other authors [2,5,7,13,14,15,19]. It can also be observed that even with an infill density lower than 100%, regardless of the type (diagonal or honeycomb), the Young’s modulus values are higher than those of injection-molded parts, which have a 100% infill. This finding contradicts other research, where strength resistance tests on samples with non-solid infill show values ranging from 60% to 80% of those of injection-molded samples [6].

In the analyzed case, the lowest Young’s modulus values were calculated for samples made from the material supplied by Company 2. No clear patterns were observed across all samples. However, for samples made from the material supplied by Company 3, it was evident that the infill density had a greater influence on the results than the infill pattern. As the infill density increased, the Young’s modulus values also increased proportionally. For materials from Companies 4 and 5, higher Young’s modulus values were observed for samples with a 60% infill density. The significant impact of the infill density on mechanical strength parameters has also been reported in previous research [3,6,7].

To assess the sensitivity of the estimated Young’s modulus to the variable factors of (1) deposition strategy and (2) manufacturer, an analysis of variance (ANOVA) was performed. The results are summarized in Table 1.

It can be observed that both factors, the manufacturing strategy and the material supplier significantly influence the estimated Young’s modulus. This is supported by the *p*-value, which is much lower than the typical significance level (alpha = 0.05) assumed in ANOVA and F-tests. Additionally, for both factors, the calculated F value exceeds the critical F value, indicating a statistically significant effect. However, no interaction was found between the two factors (“strategy” and “company”), suggesting that these factors are independent of each other. Therefore, there is no need to conduct further analysis with replication, as the factors do not show any dependency.

**Figure 4:**
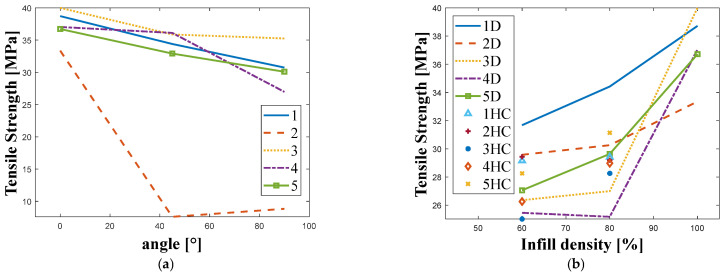
Plots of tensile strength results obtained for the tested manufacturers for: (**a**) Different manufacturing angle. (**b**) Different infill density and infill structure. ((1–5)—manufacturer; HC—honeycomb; D—diagonal).

The tensile strength results for each combination of infill density, infill pattern and orientation angle are shown in Figure 4.

Considering the manufacturing angle, it can be observed that the tensile strength of all materials was highest at 0° and decreased as the angle increased. However, it is worth noting that for Company 3′s material (Figure 4a), the values obtained were the most stable. The manufacturing angle had the greatest effect for the filament from Company 2, where a significant decrease in tensile strength was recorded for angles other than 0°. Considering the type and density of infill, it can be seen that for almost all materials, the tensile strength increased with the infill density and reached its highest value for Company 1′s material with a diagonal structure.

**Figure 5:**
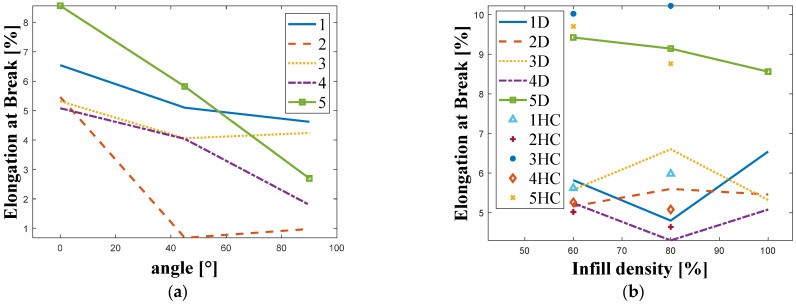
Plots of fracture elongation results obtained for the tested manufacturers for: (**a**) Different manufacturing angle. (**b**) Different infill density and infill structure. ((1–5)—manufacturer; HC—honeycomb; D—diagonal).

Figure 5 shows the relationship between elongation at break and the manufacturing angle (Figure 5a) and between the infill density and infill pattern for different manufacturers (Figure 5b).

**Figure 6:**
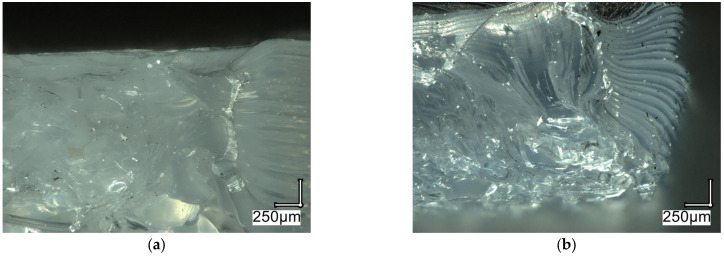
Microscopic images for samples with a infill density of 80% produced at an angle of 0° obtained for: (**a**) Company 3–honeycomb. (**b**) Company 1–diagonal. Magnification 100×.

In the case of elongation at break, a similar relationship can be observed to that for tensile strength, where the highest values were obtained at an angle of 0° and decreased as the angle increased. As before, the lowest values were recorded for Company 2, and the highest for Company 5. Taking into account the infill density and infill pattern, the highest values in both cases were recorded for the material from Company 3. Figure 6 shows examples of fracture images obtained for samples with an infill density of 80% and manufactured at an angle of 0°.

Microscopic analysis of the obtained images indicates that, in the case of the sample with a honeycomb infill, the fracture exhibited a heterogeneous, irregular character, with numerous voids and local defects between the cell walls. Such morphology suggests limited adhesion between the filament paths and local structural weaknesses, which promote premature crack initiation. For the analysed sample, lower values of Young’s modulus (974 MPa) and tensile strength (28 MPa) were obtained compared with sample b, while elongation at break was higher (10.2%). This suggests that the honeycomb structure exhibits a more ductile mode of failure, which may result from its elastic, three-dimensional geometry.

For the analyzed sample with a diagonal infill, a markedly higher Young’s modulus (1250 MPa) and greater tensile strength (34 MPa) were obtained, with a lower elongation at break (4.8%). The observed fracture exhibits a more organized and compact character, with visible directional traces of stretching and plasticization, indicating better interlayer adhesion and more efficient stress transfer along the filament paths. The lower elongation at break, however, suggests a stiffer, yet less ductile, material behavior.

**Figure 7:**
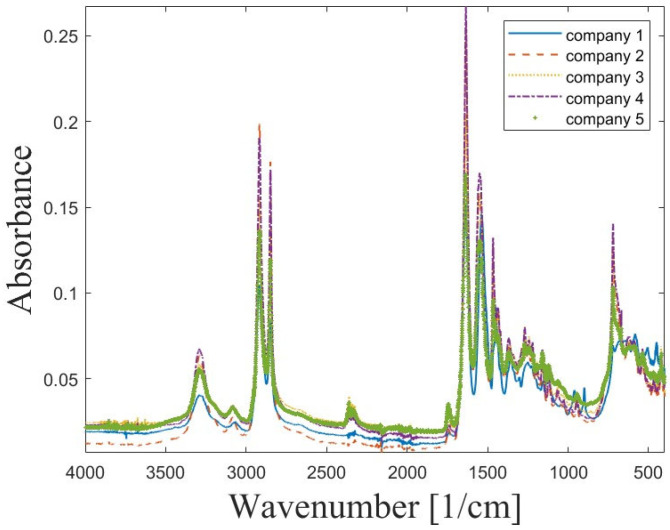
FTIR plot of samples composition.

Given the diverse results obtained for different manufacturers, FTIR analyses were conducted to identify the materials under investigation. The Fourier transform infrared (FTIR) spectra of samples are shown in Figure 7.

The original OPUS software (Bruker, Ettlingen, Germany) used for measurement and spectral analysis identified the blue plot as a type of PA6 plastic from various manufacturers. Significant peaks were numerically determined and compared with the default PA6 template to verify this observation. In contrast, the spectra for the other four materials exhibited significant peaks corresponding to the PA12 template, although the spectral bands in this key region were less intense, which is characteristic of PA12. To verify the preliminary results, a compilation of the characteristic bands for the proposed polymers was prepared (Table 2).

**Figure 8:**
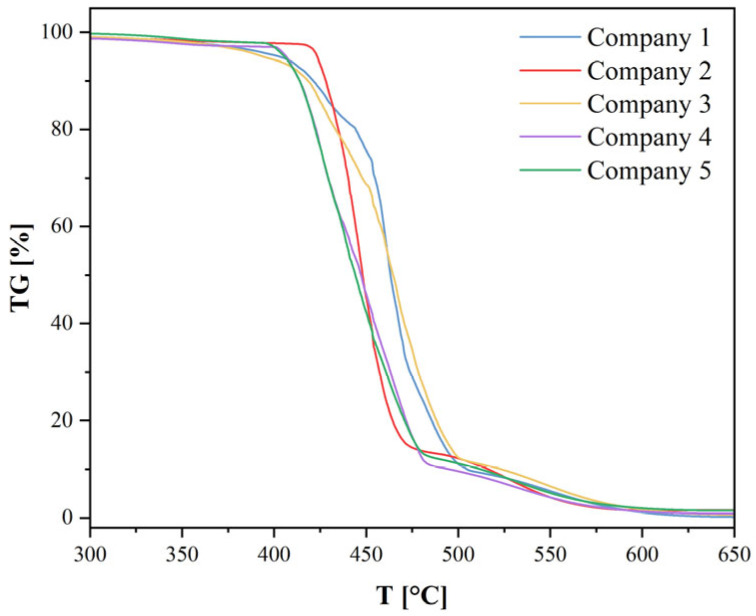
Mass loss (TG) plot of samples obtained from thermogravimetric measurements.

**Figure 9:**
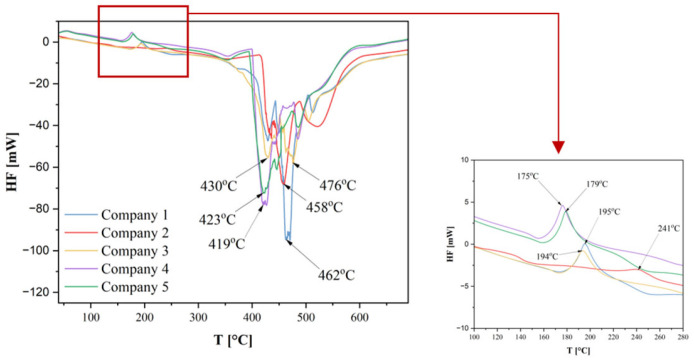
Heat flow (HF) plot of samples obtained from thermogravimetric measurements.

**Figure 10:**
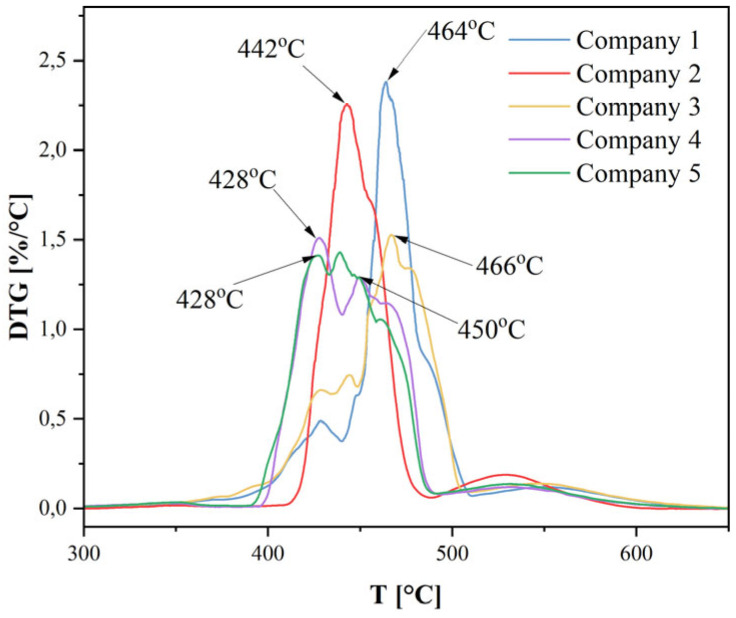
Derivative mass (DTG) plot of samples obtained from thermogravimetric measurements.

Analysing the characteristic peaks summarised in Table 2, it can be observed that their frequency bands are similar, with a slight shift towards lower wavenumbers for PA6. The differences between PA6 and PA12 described in studies [22,23,24,25,26] are not as pronounced in Figure 7. The ν(N–H) vibration recorded for the material from Company 1 exhibits lower intensity, which is typical of PA6, yet it does not differ significantly from those recorded for the other materials. A key indicator of the extended chain length in PA12 is the aliphatic index, defined as the ratio of the intensity of the C–H stretching bands (in the range 2850–2930 cm^−1^) to the Amide I (~1640 cm^−1^) or Amide II (~1545 cm^−1^) bands. In PA12, which has 11 carbon atoms per amide group, the C–H bands are considerably more intense relative to the amide bands than in PA6, which has 5 carbon atoms per amide group. Furthermore, in both polyamides, the presence of strong hydrogen bonds is confirmed by the broad Amide A band around ~3300 cm^−1^. However, the higher density of amide groups in PA6 potentially results in a stronger and more regular hydrogen-bonding network, contributing to its higher melting temperature and stiffness. The Amide IV vibration is characteristic of the crystalline phase of PA6, and in Figure 7, only one distinct peak is observed for the material from Company 1, which does not differ substantially in intensity from the spectra recorded for the other materials. For the remaining manufacturers, the more intense peak is in the CH_2_ rocking region, which is a defining band for PA12. Due to its exceptionally long alkyl segment (N=11), PA12 exhibits a strong, sharp absorption band at 719–725 cm^−1^. This corresponds to the CH_2_ rocking vibration, characteristic of long, ordered alkylene segments packed in a regular structure. The presence of this band is a decisive marker of the long alkyl chain and is characteristic of the PA12 spectrum [22,23]. Based on the FTIR spectra, it can therefore be tentatively concluded that the investigated materials are Polyamide 12 (PA12), while the observed differences may arise from the use of different additives, fillers, or variations in molecular weight. To confirm these conclusions, TG–DTG analysis was subsequently performed, with the results presented in Figure 8, Figure 9 and Figure 10.

The previously conducted FTIR analysis identified the investigated materials (Companies 1–5) as Polyamide 12 (PA12) or a polyamide with a similar structure containing a long alkyl chain, mainly based on the Amide I band and the rocking vibration peak around ~720 cm^−1^. Thermogravimetric (TG) analysis confirms this identity and also reveals differences in thermal stability and degradation mechanisms among the individual PA12-based material samples. Figure 8 presents the percentage mass loss of each sample as a function of the measured temperature for all investigated materials. The characteristic sharp mass loss in the range of approximately 400–500 °C is typical for the thermal degradation of polyamides in air. Significant mass loss for all samples begins around 410–420 °C. For pure PA12, the maximum degradation rate (T_max_, DTG) is typically observed at 438 °C [F]. This high degradation temperature is consistent with the known thermal stability of PA12. It can also be noted that after heating to 700 °C, the residual mass of each sample is very low (close to 0%), which is typical for the decomposition of pure organic polymers. The TG curves for samples from Companies 1–5 clearly confirm that these materials belong to the family of polyamides with high thermal stability, and their degradation profiles are consistent with the characteristics of Polyamide 12 (PA12). Differences among the individual TG curves suggest that these companies offer PA12 variants differing in additives (e.g., thermal stabilizers or processing aids), which slightly modify the onset temperature of degradation.

Figure 9 presents the heat flow (HF) as a function of temperature. In the low-temperature region, characteristic of PA12, a melting peak is observed around 175–185 °C [22,24,27]. For the investigated materials (Companies 1, 3–5), peaks are visible in the range of 160–220 °C, corresponding to the melting temperature (T_m_). Only for the material from Company 2 there is an absence of a significant peak in this range, indicating a very low degree of crystallinity. The amount of crystalline phase is too small to produce a noticeable endothermic heat absorption. The main thermal activity, comprising a series of sharp, deep, and varied peaks, occurs in the range of approximately 350–500 °C and corresponds to the thermal decomposition of the polyamide. This temperature range closely matches that in which mass loss was observed on the TG curve. The presence of multiple peaks indicates a complex thermal degradation kinetics and a sequence of bond scissions or additional processes resulting from the presence of additives or different types of defects in the polymer. The degradation process of the investigated materials is not purely single-step but consists of a series of overlapping thermal reactions.

Sample Company 1 exhibits the greatest depth of the endothermic peak (strongest HF signal) at around 460 °C. This suggests that it is the most ordered or purest material, requiring the greatest energy (heat) to initiate and complete the decomposition process. Smaller and less sharp endothermic peaks may indicate that the degradation process was less energetic, started earlier, and was more extended, which could be due to the presence of degradation catalysts, varied thermal stability, impurities, additives, or lower molecular weight [22,27].

The DTG curve (Figure 10) focuses on the region of thermal degradation, which in the TG curve was represented by a sharp mass loss. The DTG curves for all samples exhibit a main peak in the range of 400–480 °C. This is consistent with the high thermal stability of PA12, for which the temperature of maximum degradation (T_max_) is often reported in the literature as approximately 438 °C [F]. The DTG curve illustrates that the thermal decomposition of PA12 is not purely a single-step process, as suggested by the complex peaks observed in the HF curve. Samples 1 and 2 display a dominant, sharp, single peak, suggesting the predominance of a single main decomposition mechanism (C–C bond scission), characteristic of PA12. Their high intensity indicates a rapid and vigorous pyrolysis process. In samples 3 and 4 (and also in 5), a clear shoulder peak is visible. This indicates that these materials undergo two (or more) overlapping degradation processes with different activation energies and different temperatures of maximum rate. The earlier stage may be associated with the decomposition of less stable components (e.g., chain ends, low-molecular-weight additives, or degraded polymer fragments), whereas the later stage likely corresponds to the main thermal degradation of the PA12 polymer backbone.

## 4. Discussion

Liu Z. et al. [28] studied the impact of model orientation, raster angle, and filler type on the strength properties of PLA samples. They found that the mechanical properties were significantly influenced not only by the filling materials but also by the printing orientation and raster angle. For instance, in a flat orientation, the tensile modulus and strength were higher when layers were deposited at (+45°/−45°) compared to (0°/90°) deposition. However, their results differ from those obtained in the present study—where the highest Young’s modulus values for PA12 were recorded at 0° orientation. This suggests that FFF process parameters may have varying effects depending on the material used.

Similar results to those presented here were reported by Palma T. et al. [29], who examined the effect of build orientation on PA12 using Multi Jet Fusion (MJF) technology. Their mechanical characterization showed that samples manufactured in a vertical orientation exhibited lower values of elastic modulus, hardness, and wear resistance, compared to horizontally printed parts.

Mostafa K.G. et al. [7] investigated the influence of seven FFF process parameters on the ultimate tensile and flexural strength, as well as the modulus of elasticity, building time, and volume of 3D printed Nylon 12™, using Taguchi’s L18 orthogonal array. They found that the most significant parameter affecting material properties and production costs was infill density, with the ability to control this parameter accounting for approximately 80% of the observed effects. This is consistent with the results of this study, where the infill density (Table A1, Figure 7) was shown to have a significant effect on the strength properties.

Popescu D. et al. [2], in their literature review, confirmed that both infill density and build orientation influence mechanical properties, a finding echoed by Sheoran A.J. [15]. Their work also indicated that a build angle/orientation of 0° leads to the highest strength properties in FFF-processed parts, which aligns with the results presented here. Dudescu C. et al. [2] further explored the influence of printing direction, infill density, and pattern on the tensile strength of ABS samples. They observed that Young’s modulus increased with infill density percentage, although the relationship was non-linear, with diminishing returns at higher infill densities.

The build orientation and printing parameters (such as nozzle temperature, printing speed, layer thickness, and infill pattern) directly affect the quality of bonding between adjacent paths and layers of the molten material. This effect has been observed experimentally, for example, by Vaes et al. [30], who linked interlayer weld strength to thermal history and polymer chain diffusion in semi-crystalline polymers. Similarly, Cunha & Robbins [31] highlighted that flow-induced molecular alignment and chain disentanglement strongly influence the welding and mechanical strength of polymer interfaces in FFF.

The mechanical strength of FFF-fabricated components primarily depends on the degree of polymer chain diffusion across layer boundaries, as well as on the amount and distribution of pores formed due to insufficient wetting and cooling of the material. In cases where stresses act perpendicular to the layer direction, the interlayer bonds represent the weakest link in the structure, leading to a reduction in both strength and elastic modulus, as also demonstrated by Vaes et al. [30].

Conversely, optimization of build orientation and process parameters allows for balancing production time and cost with the required structural quality and mechanical performance, as discussed in Shaqour et al. [32]. To accurately determine the optimal compromise, it is necessary to move from phenomenological observation to integrated computational modelling. Such an approach would require coupling thermal finite element analysis (FEA) with constitutive models based on polymer chain diffusion/reptation theory. Applying this integrated approach would enable accurate determination of the maximum deposition speed (or other process rates) that ensures the required G_IC_ threshold for a given build orientation, providing a scientific foundation for effectively balancing cost and performance in FFF manufacturing using PA12 or similar engineering thermoplastics.

Based on the reviewed literature and the present findings, it can be concluded that optimizing build orientation and process parameters plays a crucial role in balancing production costs and achieving desired mechanical properties for 3D printed parts.

## 5. Conclusions

The results of this study indicate that both the deposition parameters and the origin of the filament (i.e., the manufacturer) significantly influence the mechanical properties of PA12 samples produced using the FFF process. Among the process parameters, the highest Young’s modulus values were obtained for samples with a 100% infill density, deposited on the machine bed at a 0° orientation.

Analysis of different infill densities and patterns revealed that variations in infill structure and lower infill densities (60% and 80%) only had a minor effect on the mechanical properties. The results for these configurations were generally consistent. However, notable differences were observed based on the filament supplier. In most cases (6 out of 7), the lowest Young’s modulus values were recorded for material from Company 2, while the highest values were observed for materials from Companies 3 and 4 (in 3 out of 7 cases, respectively).

The data suggest that 3D printing using the FFF process has the potential to modify the mechanical properties of PA12, particularly enhancing its stiffness. This trend was especially evident for materials from Companies 2 and 4, particularly with a 60% honeycomb infill. Furthermore, even for the 45° printing angle, where layer misalignment occurs and cross-sectional dimensions between layers vary, an increase in Young’s modulus was still observed, highlighting the complex interplay of these factors.

To examine more precisely the discrepancies in Young’s modulus values among the filament manufacturers, FTIR analysis was performed, followed by TG–DTG analysis. The results confirmed that the investigated materials were polyamides, suggesting that in each case the material was PA12. The deviations observed in the curves obtained from both analyses may have been caused by different types of additives used by the manufacturers, which could also be reflected in the mechanical test results.

Finally, the ANOVA without replication test demonstrated that both the FFF process parameters and the material manufacturer have a significant influence on the Young’s modulus of the printed parts.

Future research should focus on investigating the long-term mechanical performance of FFF-printed PA12 parts, particularly under dynamic or cyclic loading conditions, to better predict their suitability for real-world applications. Additionally, further studies could explore the development of optimized process parameters tailored to specific manufacturing needs, such as in medical or aerospace applications.

## Figures and Tables

**Table 1:** Two-factor ANOVA–“Company” (Rows) & “Strategy” (columns).

*Variance Source*	*SS*	*df*	*MS*	*F*	*p-Value*	*Test F*
Rows-company	4,832,668	24	201,361.2	4.715119	1.75 × 10^−9^	1.593228
Columns-strategy	2,635,365	6	439,227.4	10.28505	1.82 × 10^−9^	2.162091
Deviation	6,149,581	144	42,705.42			
Sum	13,617,614	174				

**Table 2:** Characteristic infrared bands and their assignments of filament samples.

Vibrations	Functional Group Assignment	Frequency [cm^−1^]		Intensity
		PA6	PA12	
ν(N–H)	Stretching (hydrogen bonded)	3289–3292	3300–3320	Strong (S)
ν_a_(CH_2_)	Asymmetric stretching CH_2_	2921–2926	2915–2925	Medium (M)
ν_s_(CH_2_)	Symmetric stretching CH_2_	2853–2855	2845–2855	Medium (M)
Amide I	ν(C=O) Stretching	1630–1633	1640–1650	Very Strong (VS)
Amide II	δ(N–H) Bending + ν(C–N) Stretching	1541–1543	1545–1555	Strong (S)
δ(CH_2_)	Bending CH_2_	1460–1465	1470–1475	Medium (M)
Amide III	ν(C–N) Stretching	1200–1270	1200–1250	Medium (M)
Amide IV	δ(N–H) Out of plane bending	930–970	950–990	Weak (W)
φ(CH_2_)	Rocking CH_2_	Weak or absent	719–725	Strong (S)

## Data Availability

The original contributions presented in this study are included in the article. Further inquiries can be directed to the corresponding author.

## Appendix A

**Table A1:** List of determined longitudinal elasticity modules (Young’s modulus - E), tensile strength and elongation at break of polyamide samples obtained from tensile strength tests.

Manufacturer	Infill [%]	Infill Pattern	Angle [°]	E [MPa]	St. Dev.*	Tensile Strength [MPa]	St. Dev.*	Elongation at Break [%]	St. Dev.*
References	100	-	-	1023	100	58	3.5	283	23.8
Company 1	100	Diagonal	0	1520	330	39	1.4	6.5	1.6
	100	Diagonal	45	1104	420	34	0.7	5.1	0.6
	100	Diagonal	90	1130	242	31	1.6	4.6	1.5
	80	Diagonal	0	1250	95	34	1.0	4.8	0.2
	60	Diagonal	0	1097	76	32	1.0	5.8	0.7
	80	Honneycomb	0	1157	179	29	1.4	6.0	0.9
	60	Honneycomb	0	1131	50	29	1.4	5.6	0.7
Company 2	100	Diagonal	0	967	170	33	0.8	5.5	0.4
	100	Diagonal	45	682	39	7.6	0.5	0.7	0
	100	Diagonal	90	571	38	8.9	1.4	1.0	0.1
	80	Diagonal	0	865	137	30	0.9	5.6	0.2
	60	Diagonal	0	731	94	30	1.1	5.2	0.4
	80	Honneycomb	0	850	101	29	0.7	4.6	0.2
	60	Honneycomb	0	814	82	29	1.3	5.0	0.3
Company 3	100	Diagonal	0	1800	106	40	0.9	5.3	0.5
	100	Diagonal	45	1407	104	36	1.6	4.0	0.4
	100	Diagonal	90	1149	83	35	0.5	4.2	0.3
	80	Diagonal	0	1116	103	27	1.3	6.6	1.2
	60	Diagonal	0	978	88	26	1.7	5.6	1.0
	80	Honneycomb	0	974	415	28	1.7	10.2	1.4
	60	Honneycomb	0	1097	116	25	1.1	10.0	1.4
Company 4	100	Diagonal	0	1297	116	37	0.5	5.1	0.6
	100	Diagonal	45	1350	101	26	1.8	4.0	0.4
	100	Diagonal	90	1352	142	27	8.6	1.8	0.9
	80	Diagonal	0	1059	166	25	1.3	4.3	0.5
	60	Diagonal	0	954	122	26	1.5	5.2	1.4
	80	Honneycomb	0	1064	118	29	4.4	5.1	0.7
	60	Honneycomb	0	1041	174	26	2.1	5.3	0.6
Company 5	100	Diagonal	0	1159	166	37	0.7	8.6	0.8
	100	Diagonal	45	1001	207	33	0.4	5.9	0.7
	100	Diagonal	90	1074	53	30	3.1	2.7	0.6
	80	Diagonal	0	967	81	30	0.3	9.1	0.7
	60	Diagonal	0	949	152	27	0.4	9.4	0.7
	80	Honneycomb	0	1103	29	31	0.9	8.8	0.6
	60	Honneycomb	0	960	67	28	0.6	9.7	0.4

* St. Dev.–Standard deviation.
